# Reply to: ‘Predicted loss-of-function variants before Met584 in *ARID1B* in population cohorts likely reflect reduced penetrance and should be reported diagnostically’

**DOI:** 10.1186/s13073-025-01544-3

**Published:** 2025-10-07

**Authors:** Caroline F. Wright, Robin N. Beaumont

**Affiliations:** Department of Clinical and Biomedical Sciences, University of Exeter Medical School, Garden Suite, St Luke’s Campus, Magdalen Road, Exeter, EX1 2LU UK

We appreciate the careful consideration given by van der Sluijs and Santen of our work comparing clustering of predicted loss-of-function (pLoF) variants in clinical versus population cohorts [1]. Although they largely agree with our findings, they have provided evidence that pLoF variants in *ARID1B* (associated with Coffin-Siris syndrome, MIM #135900) can be pathogenic even where they occur early in the gene. This contrasts with our finding that the majority of pLoF variants in UK Biobank were in the first quarter of the gene and were unlikely to be pathogenic. We hypothesized that this clustering was due to the presence of an alternative start site at p.Met584 of the MANE Select transcript, which is near the start of several alternative transcripts and corresponds to low expression of the first quarter of the gene [2]. However, very few rules in biology are absolute and there are likely to be exceptions, as shown conclusively by van der Sluijs and Santen through the use of orthogonal functional validation to prove the pathogenicity of pLoF variants in this region. Nonetheless, we believe our original assertion that these variants “should not be routinely reported diagnostically” is still appropriate based on consideration of wider population and clinical data.

Firstly, it is notable that only ~ 10% of pathogenic/likely pathogenic pLoF variants in DECIPHER or ClinVar occur in the first ~ 25% of the protein and before Met584 (Fig. 1) [3, 4]. Interestingly, the majority of these variants—including those reported by van der Sluijs and Santen—are insertions/deletions near Met584 itself that are likely to reduce its viability as a functional start codon. Secondly, in stark contrast, ~ 82% of pLoF variant carriers (~ 87% excluding splice variants) in UK Biobank occur in the first ~ 25% of the protein. Importantly, there are no significant burden associations between *ARID1B* pLoF variants and any relevant phenotypes in UK Biobank [5, 6], supporting the fact that these are likely benign variants. Therefore, in the absence of additional evidence such as a supportive episignature, these variants should be treated with caution in a clinical context.

**Fig. 1:**
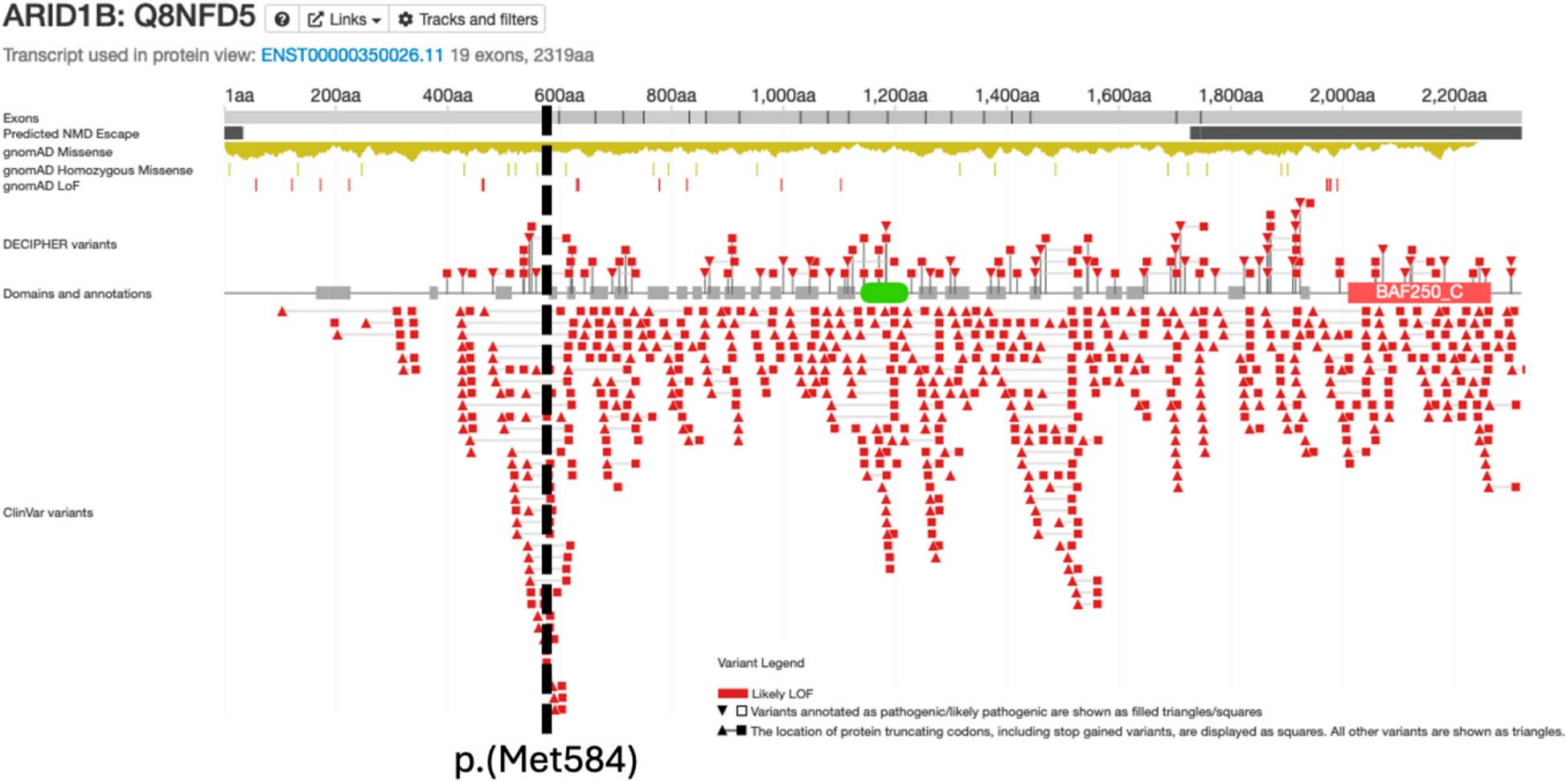
Distribution of pathogenic/likely pathogenic pLoF variants in *ARID1B*. Location of the alternative start site at Met584 is highlighted; several alternative transcripts start within a few amino acids of this position and just prior to the end of the large first exon. Figure used with permission and adapted from DECIPHER (12 February 2025) [3]: https://www.deciphergenomics.org/gene/ARID1B/overview/protein-genomic-info

Examples such as this are invaluable for highlighting the importance of both large population and clinical datasets as well as functional validation to inform clinical variant interpretation. They also raise wider philosophical questions about when variants should be classified as pathogenic with incomplete penetrance rather than being considered risk factors. Ultimately, careful curation and consideration of context are critical for accurate and safe variant interpretation.

## Data Availability

No datasets were generated or analyzed during the current study.
